# Antenatal Training with Music and Maternal Talk Concurrently May Reduce Autistic-Like Behaviors at around 3 Years of Age

**DOI:** 10.3389/fpsyt.2017.00305

**Published:** 2018-01-11

**Authors:** Zeng-Liang Ruan, Li Liu, Esben Strodl, Li-Jun Fan, Xiao-Na Yin, Guo-Min Wen, Deng-Li Sun, Dan-Xia Xian, Hui Jiang, Jin Jing, Yu Jin, Chuan-An Wu, Wei-Qing Chen

**Affiliations:** ^1^Department of Biostatistics and Epidemiology, School of Public Health, Sun Yat-sen University, Guangzhou, China; ^2^School of Psychology and Counselling, Queensland University of Technology, Brisbane, QLD, Australia; ^3^Women’s and Children’s Hospital of Longhua District of Shenzhen, Shenzhen, China; ^4^Department of Maternal and Child Health, School of Public Health, Sun Yat-sen University, Guangzhou, China; ^5^Department of Information Management, Xinhua College of Sun Yat-sen University, Guangzhou, China

**Keywords:** antenatal training, prenatal stimulation, fetus music, maternal voice, autistic-like behavior, neurobehavioral development

## Abstract

Antenatal training through music and maternal talk to the unborn fetus is a topic of general interest for parents-to-be in China, but we still lack a comprehensive assessment of their effects on the development of autistic-like behaviors during early childhood. During 2014–2016, 34,749 parents of children around the age of 3 years who were enrolled at kindergarten in the Longhua district of Shenzhen participated in this study. Self-administered questionnaires regarding demographics, antenatal music training, and maternal talk to the fetus during pregnancy were completed by the children’s primary caregivers. Autistic-like behaviors were assessed using the Autism Behavioral Checklist. Tobit regression analyses revealed that antenatal music training and maternal talk to the fetus was associated with a reduction in autistic-like behaviors in children, with a dose-dependent relationship. Furthermore, factorial analysis of covariance indicated a significant interaction effect between antenatal music training and maternal talk to the fetus on the autistic-like behaviors and found that children who often experienced antenatal music training and maternal talk concurrently had the lowest risk of autistic-like behaviors, while children who were never exposed to maternal talk and only sometimes experienced antenatal music training had the highest risk. Our results suggest that antenatal training through both music and maternal talk to the unborn fetus might reduce the risk of children’s autistic-like behaviors at around 3 years of age.

## Introduction

Autism spectrum disorder (ASD) is a spectrum of neurodevelopmental disorders characterized by deficits in social communication and social interaction along with restricted interests and repetitive or stereotyped patterns of behaviors (1). Symptoms of ASD may be seen in the first year of life, but are typically recognized in the second year and are often not diagnosed until the age of 3 years (1, 2), which is the age requirement for kindergarten entrance in China. ASD not only has an early onset in the developmental period (especially in the preschool age), but also has a tendency to persist into adulthood (3). Based on 2014 data from the Centers for Disease Control and Prevention, ASD afflicted 2.24% of children aged 3–17 years in the United States, and its prevalence increased steadily (4, 5). In Asian countries, recent surveys revealed that the prevalence of ASD were 0.25, 0.23, 1.81, and 2.64% for China, India, Japan, and Korea, respectively (2, 6–8). Moreover, a majority of children with ASD were male, with a variable female-to-male ratio ranging from 1:16.0 to 1:1.33 (9). In addition to relatively high prevalence rates, ASD can impair quality of life and lead to a high cost for families and society. In 2010, the total estimated European cost of ASD was €2,546 million, a figure which did not take into account indirect costs such as the loss of school time and difficulties with academic performance (10). As such, internationally there is a significant need to better understand the risk factors for the onset of ASD in order to guide the development of effective public health interventions for the onset of ASD.

Although ASD is usually considered a clinical condition, a wide spectrum of autistic-like behaviors, such as impairment in social communication, may exist across the general population (11, 12). Risk factors (e.g., paternal age at birth) implicated in trajectories leading to ASD have also been shown to associate with the appearance of autistic-like behaviors in a study built on two national representative cohorts of twins from Sweden and the United Kingdom (13). Another study confirmed an etiological similarity in both ASD and autistic-like behaviors, which suggested that they were different from each other only in the severity of functional impairment (14). Moreover, ASD and autistic-like behaviors have similar patterns of comorbidity. For example, they are strongly related to conduct problems and attention-deficit/hyperactivity disorder (15, 16). It has been well documented that individuals exhibiting sub-threshold levels of autistic-like behaviors also display other cognitive and social problems. For instance, nonclinical samples of individuals with autistic-like behaviors also displayed problems such as socio-cognitive difficulties, communication obstacles, and sensory processing problems (17, 18). Moreover, those with more or less autistic-like behaviors had different white matter volumes in the posterior superior temporal sulcus, a brain region important for dealing with socially related impulses and which has been associated with functional deficits in ASD (19). Therefore, as reported by Constantino and Todd, given the continuous distribution of autistic-like behaviors in the general population, it may be arbitrary to distinguish between clinical and subclinical levels of such disorders (20) and, therefore, also important to investigate the risk factors for the onset of autistic-like behaviors.

There is emerging evidence that auditory stimulation of an *in utero* human fetus is a predictor for a range of positive outcomes for both the mother and the fetus/child. For instance, mother–fetus interactions through maternal talk and tactual stimulation are effective in enhancing mother–fetus attachment and improving maternal health relevant coping behaviors during pregnancy (21, 22). Moreover, a study has showed that a fetus can hear music in the womb as early as 16 weeks of gestation and responded directly to it by opening their mouths and moving their tongues (23). Fetal sound exposure in the womb, such as talking and heartbeats, may influence their auditory preferences (24). Neonates can display a preference for their own mother’s voice as soon as several hours after birth, which indicates prenatal origins for language abilities and provides the evidence of fetal learning and memory of voice and language in the womb (24, 25). Furthermore, a fetus can memorize not only his mother’s voice but also more complexly external noises, and these sounds may have important effects on the development of the nervous system (26–28). These findings suggest that certain types of auditory stimulation of an *in utero* human fetus may offer protective or even enhancing effects upon children’s neurobiological outcomes.

Antenatal training has been defined as the process whereby women employ various auditory stimulations, such as playing music, talking to their fetus, and belly touching during pregnancy, to encourage beneficial effects on birth outcomes and children’s development (29, 30). In particular, antenatal music training is defined as when the pregnant women uses music, especially certain kinds of positive music (such as classical music, nursery rhymes, lullabies, etc.), to stimulate the fetus with the expectation that the basic elements of the music (e.g., harmony, melody, and rhythm) could have a positive effect upon the fetus. Such practices have a long history in countries such as China. As far as records can indicate, antenatal training was originally performed in Yin and Zhou Dynasty of China over 3,000 years ago (31). In modern China, playing music and maternal talk to the fetus are the most frequently used practices of antenatal training. These two practices are widely adopted by pregnant women in China to communicate with their fetus and have been shown to bring about many beneficial effects on fetal and neonatal development (32, 33). However, the question of how antenatal music training and maternal talk to a fetus affects the development of autistic-like behaviors in young children has never been explored. We, therefore, aimed to examine these relationships and hypothesized that antenatal training with music and maternal talk to the fetus will have a positive impact upon young children’s neuropsychological and psychiatric development based on DOHaD model, which proposes a link between fetal development and non-communicable diseases emerging in adulthood (34, 35). In particular, we hypothesized that children of mothers who report using antenatal music training during pregnancy or mothers who report talking more frequently with their fetus while pregnant would exhibit a lower risk of autistic-like behaviors around the age of 3 years.

## Materials and Methods

### Study Population

The Longhua Child Cohort Study (LCCS) was set up to research the impacts of family and school environment on children’s mental health and abnormal behaviors, including autistic-like behaviors, conduct problems, and hyperactivity behaviors (36–38). The baseline survey was conducted in the years from 2014 to 2016. A total of 40,273 children aged around 3 years were enrolled when they entered the 171 kindergartens in Longhua District of Shenzhen, China. This study was approved by the Ethic Committee of School of Public Health of Sun Yat-sen University. Written informed consent was obtained from all parents or guardians of children who took part in the study, in accordance with the Declaration of Helsinki.

### Data Collection

The children’s primary caregivers were asked to complete a self-administered structured questionnaire regarding the parents’ socio-demographic characteristics (including marital status, age at birth of the study child, family income, and education level), frequencies of antenatal music training, and maternal talk to fetus during pregnancy, as well as information about their Children (such as birth date, sex, and autistic-like behaviors).

### Measurement of Antenatal Training

Antenatal music training and maternal talk to the fetus were assessed by two questions: “*How often did the mother perform antenatal training through music stimulation during pregnancy*,” and “*How often did the mother perform antenatal training through talking to the fetus during pregnancy*.” Both antenatal music training and maternal talk to the fetus were rated on a four-point Likert scale: never, occasionally (<1 time per week), sometimes (1–4 times per week), and often (>4 times per week).

### Measurement of Autistic-Like Behaviors

The children’s autistic-like behaviors were measured using the Autism Behavior Checklist [ABC; (39)]. The ABC is an assessment instrument designed to screen autism in children, utilizing an observer’s rating of the child’s behavior to quantify behaviors typically associated with autism. The scale has been widely used internationally since its development in 1980 and has been shown to have good predictive validity in screening autistic children (40). The adapted Chinese version of ABC is widely used in Mainland China, and has been shown to have a good interrater reliability of 0.785 and a good test–retest reliability of 0.789, as well as having good predictive validity in screening autism among Chinese children (41). Given these good psychometric properties, the ABC was chosen as a measure of autistic-like behaviors in this study. In accordance with published guidelines, each of the 57 items were rated on a scale from 1 to 4 according to its relevance to autism, and these items were grouped into five aspects: Sensory, Relating, Body and Object Use, Language, and Social and Self-Help Skills (40). The total ABC score was calculated by adding the scores of all items and ranged from 0 to 158 (with higher scores indicating more autistic-like behaviors). A standard cutoff value of 67 was used as the threshold of a high probability of ASD (40).

### Covariates

The covariates included mother’s marital status (married, divorced, widowed, and unmarried), age at the time of the child’s birth, education level (less than high school, high school, college, and postgraduate), gestational diseases (including gestational diabetes mellitus, preeclampsia/eclampsia, and gestational hypertension), family income (≤5,000, 5,000–10,000, 10,000–15,000, and >15,000 RMB per year) and the child’s sex.

### Statistical Analysis

Categorical and ordinal variables were described as absolute frequencies and proportions, while continuous variables as mean (SD) or median (quartile), depending on normal or skewed distribution. Specifically, the ordinal data of antenatal music training and maternal talk were coded as 0 (never), 1 (occasionally), 2 (sometimes), and 3 (often), and then the categorical variables were transformed into dummy variables before the application of further analyses. Due to a large number of 0 values, the ABC score of each child was added “1” and then the new score was transformed using natural log (ln) transformation to reduce skewness.

The association of antenatal music training and maternal talk to the fetus with autistic-like behaviors was separately analyzed using Tobit regression models with or without adjusting for the aforementioned covariates, and coefficients, and SE were used to express their strengths of association and variation. In addition, means and 95% confidence intervals (CIs) of percentage changes of “ABC score + 1” in different frequency groups of antenatal music training and maternal talk to fetus were obtained by exponentiating the natural log scaled beta coefficient of Tobit regression with adjusting for the covariates and subtracting 1. The calculation of percentage change from the log scaled beta coefficient has been used in many studies before (42, 43), and its process is as follows:

(1) ln *Y* = β_0_ + β_1_*X*_1_ + β_2_*X*_2_ + … + β*_k_*X*_k_*, and ln *Y*′ = β_0_ + β_1_(*X*_1_ + 1) + β_2_*X*_2_ + … + β*_k_X_k_*;
(2) ln *Y*′ − ln *Y* = β_0_ + β_1_(*X*_1_ + 1) + β_2_*X*_2_ + … + β*_k_*X*_k_* − (β_0_ + β_1_*X*_1_ + β_2_*X*_2_ + … + β*_k_X_k_*) = β_1_;
(3) $\text{ln}\left(\frac{Y\prime }{Y}\right)=\beta_{\text{1}};$
(4) $\frac{Y\prime }{Y}={\text{e}}^{\beta }{\;}^{\text{1}};$
(5) $\frac{Y\prime -Y}{Y}={\text{e}}^{\beta }{\;}^{\text{1}}-\text{1;}$
(6) Percentage change of *Y* = e^β1^ − 1;

Moreover, we assessed potential effect modification between antenatal music training and maternal talk to the fetus, by adding a cross-product term in Tobit regression analysis with adjusting for the covariates we mentioned above. After that, factorial analysis of covariance (ANCOVA) was performed to examine main and interaction effects of antenatal music training and maternal talk to fetus on autistic-like behaviors, as well as to calculate the adjusted means of ln(ABC score + 1) among different levels of antenatal music training and maternal talk to fetus. Bonferroni correction was used for *post hoc* multiple comparisons between different groups.

The statistical analyses were performed using Statistics Analysis System (SAS, version 9.3, SAS Institute Inc., Cary, NC, USA) and R version 3.3.3 (R Foundation for Statistical Computing, Vienna, Austria), and a two-tailed *p*-value below 0.05 was considered statistically significant.

## Results

### Subject Characteristics

The characteristics of the participants are shown in Table 1. Of the 40,237 children enrolled in the kindergartens between 2014 and 2016, 5,488 (13.64%) did not complete all the questionnaire items, and so, only the remaining 34,749 (86.36%) children were included in the final data analyses. There were more boys (18,946, 54.52%) than girls (15,803, 45.48%). The mean age of participating children was 3.44 (SD = 0.54) years, and the mean maternal age at child birth was 27.38 (SD = 3.94).

**Table 1:** Characteristics of the participants.^a^

Characteristic	Mean ± SD or *n* (%) (*N* = 34,749)
Maternal age at child birth	27.379 ± 3.941
**Child sex**	
Male	18,946 (54.52)
Female	15,803 (45.48)
**Mother’s marital status**	
Married	33,946 (97.69)
Unmarried/Divorced/Widowed	803 (2.31)
**Maternal education level**	
Less than high school	5,720 (16.46)
High school	10,193 (29.33)
College	17,926 (51.59)
Postgraduate	910 (2.62)
**Family income, RMB/year**	
≤5,000	4,261 (12.26)
10,000	9,264 (26.66)
10,001–15,000	7,083 (20.38)
>15,000	14,141 (40.69)
**Gestational hypertension**	
No	34,056 (98.01)
Yes	693 (1.99)
**Preeclampsia/eclampsia**	
No	34,600 (99.57)
Yes	149 (0.43)
**Gestational diabetes mellitus**		
No	33,435 (96.22)
Yes	1,314 (3.78)
**Antenatal music training**	
Never	2,099 (6.04)
Occasionally	15,735 (45.28)
Sometimes	11,832 (34.05)
Often	5,083 (14.63)
**Maternal talk to fetus**	
Never	2,718 (7.82)
Occasionally	14,008 (40.31)
Sometimes	11,825 (34.03)
Often	6,198 (17.84)

*^a^Mean ± SD or *n* (%) are presented*.

### Autistic-Like Behaviors and the Frequency of Antenatal Music Training and Maternal Talk to Fetus in 34,749 Children

The parent-rated ABC total scores ranged from 0 to 158 in this study, with 10, 25, 50, 75, and 90% quantiles being 0, 0, 2, 10, and 22, respectively. Moreover, 227 (0.65%) subjects were over the threshold of a high probability of ASD according to the standard cutoff value of 67. Regarding the frequency of antenatal music training and maternal talk to fetus (Table 2), 1,420 (4.09%) children had the experience of only receiving antenatal music training, 801 (2.31%) children had the experience of only receiving maternal talk while a fetus, while 31,230 (89.87%) children had both antenatal music training and maternal talk while a fetus. More specifically, 10,048 (28.92%) of the children were occasionally exposed to both antenatal music training and maternal talk as a fetus, while 6,758 (19.48%) children sometimes experienced both antenatal music training and maternal talk while a fetus. However, only 34 (0.10%) children were in the group that often experienced antenatal music training but never experienced maternal talk to fetus, and only 49 (0.14%) children often experienced maternal talk as a fetus but never experienced antenatal music training.

**Table 2:** The frequency of antenatal music training and maternal talk to fetus in 34,749 children.^a^

Maternal talk to fetus	Antenatal music training				Total
	Never	Occasionally	Sometimes	Often	
Never	1,298 (3.74)	1,205 (3.47)	181 (0.52)	34 (0.10)	2,718 (7.82)
Occasionally	623 (1.79)	10,048 (28.92)	2,878 (8.28)	459 (1.32)	14,008 (40.31)
Sometimes	129 (0.37)	3,619 (10.41)	6,758 (19.45)	1,319 (3.80)	11,825 (34.03)
Often	49 (0.14)	863 (2.48)	2,015 (5.80)	3,271 (9.41)	6,198 (17.84)

Total	2,099 (6.04)	15,735 (45.28)	11,832 (34.05)	5,083 (14.63)	34,749 (100.0)

*^a^*N* (%) are presented*.

### Associations of Antenatal Music Training and Maternal Talk to Fetus with Autistic-Like Behaviors at around 3 Years of Age

Table 3 are the results derived from the Tobit regression models that measured the associations of antenatal music training or maternal talk to the fetus on autistic-like behaviors of the 3-year-old child. The results are presented for both the total ABC scores and the subscale scores. In terms of the total ABC scores, compared to the children who never experienced antenatal music training while a fetus, those who experienced antenatal training sometimes and often had a significantly lower level of autistic-like behaviors at the age of 3 years with a dose-dependent reduction in the total ABC score. This finding was replicated when also adjusting for covariates. Similarly, when compared to the children who never experienced their mother talking to them while a fetus, those who experienced maternal talk occasionally, sometimes and often has significantly lower levels of autistic-like behaviors at the age of 3 years with a dose-dependent reduction in the total ABC score. Again this finding was replicated when adjusting for the covariates used in this study.

**Table 3:** Associations of different quantities of antenatal music training and maternal talk to fetus with ln(ABC score + 1) around the age of 3 years.^a^^,^^b^

	Crude model			Adjusted model		
	Coefficient	SE	*p*-Value	Coefficient	SE	*p*-Value
**Sensory**						

**Antenatal music training**						
Never	0	–	–	0	–	–
Occasionally	−0.152	0.079	0.053	−0.036	0.079	0.646
Sometimes	−0.386	0.081	<0.0001	−0.188	0.082	0.021
Often	−0.413	0.089	<0.0001	−0.167	0.091	0.066
**Maternal talk to fetus**						
Never	0	–	–	0	–	–
Occasionally	−0.219	0.070	0.002	−0.118	0.070	0.092
Sometimes	−0.505	0.071	<0.0001	−0.316	0.073	<0.0001
Often	−0.760	0.079	<0.0001	−0.514	0.081	<0.0001

**Relating**						

**Antenatal music training**						
Never	0	–	–	0	–	–
Occasionally	−0.137	0.078	0.079	−0.032	0.079	0.688
Sometimes	−0.379	0.080	<0.0001	−0.196	0.081	0.016
Often	−0.525	0.089	<0.0001	−0.295	0.091	0.001
**Maternal talk to fetus**						
Never	0	–	–	0	–	–
Occasionally	−0.351	0.069	<0.0001	−0.260	0.069	0.0002
Sometimes	−0.579	0.071	<0.0001	−0.408	0.072	<0.0001
Often	−0.959	0.079	<0.0001	−0.732	0.080	<0.0001

**Body and object use**						

**Antenatal music training**						
Never	0	–	–	0	–	–
Occasionally	0.017	0.058	0.771	0.018	0.058	0.752
Sometimes	0.005	0.059	0.939	0.026	0.060	0.660
Often	−0.014	0.064	0.828	0.019	0.066	0.777
**Maternal talk to fetus**						
Never	0	–	–	0	–	–
Occasionally	−0.135	0.051	0.008	−0.137	0.052	0.008
Sometimes	−0.180	0.052	0.0005	−0.170	0.053	0.001
Often	−0.305	0.057	<0.0001	−0.283	0.058	<0.0001

**Language**						

**Antenatal music training**						
Never	0	–	–	0	–	–
Occasionally	−0.156	0.064	0.015	−0.053	0.064	0.410
Sometimes	−0.355	0.066	<0.0001	−0.171	0.066	0.010
Often	−0.497	0.073	<0.0001	−0.266	0.074	0.0003
**Maternal talk to fetus**						
Never	0	–	–	0	–	–
Occasionally	−0.273	0.056	<0.0001	−0.186	0.056	0.001
Sometimes	−0.606	0.058	<0.0001	−0.439	0.059	<0.0001
Often	−0.914	0.064	<0.0001	−0.694	0.065	<0.0001

**Social and self-help skills**						

**Antenatal music training**						
Never	0	–	–	0	–	–
Occasionally	−0.057	0.045	0.207	−0.053	0.045	0.243
Sometimes	−0.180	0.046	0.0001	−0.149	0.047	0.001
Often	−0.276	0.051	<0.0001	−0.231	0.052	<0.0001
**Maternal talk to fetus**						
Never	0	–	–	0	–	–
Occasionally	−0.171	0.040	<0.0001	−0.168	0.040	<0.0001
Sometimes	−0.303	0.041	<0.0001	−0.280	0.042	<0.0001
Often	−0.488	0.045	<0.0001	−0.447	0.046	<0.0001

**Total ABC score**						

**Antenatal music training**						
Never	0	–	–	0	–	–
Occasionally	−0.032	0.050	0.522	−0.0004	0.050	0.993
Sometimes	−0.193	0.051	0.0001	−0.121	0.052	0.019
Often	−0.264	0.056	<0.0001	−0.168	0.057	0.003
**Maternal talk to fetus**						
Never	0	–	–	0	–	–
Occasionally	−0.191	0.045	<0.0001	−0.166	0.045	0.0002
Sometimes	−0.383	0.045	<0.0001	−0.322	0.046	<0.0001
Often	−0.597	0.049	<0.0001	−0.507	0.050	<0.0001

*^a^Tobit regression analyses adjusted for family income, children’s sex and mother’s marital status, education level, age at birth of the study child and gestational diseases*.

*^b^β coefficients of Tobit regression on the natural log scale can be interpreted as percentage changes in original ABC scores obtained by exponentiating the coefficient and subtracting 1*.

*ABC, autism behavior checklist*.

**Figure 1:**
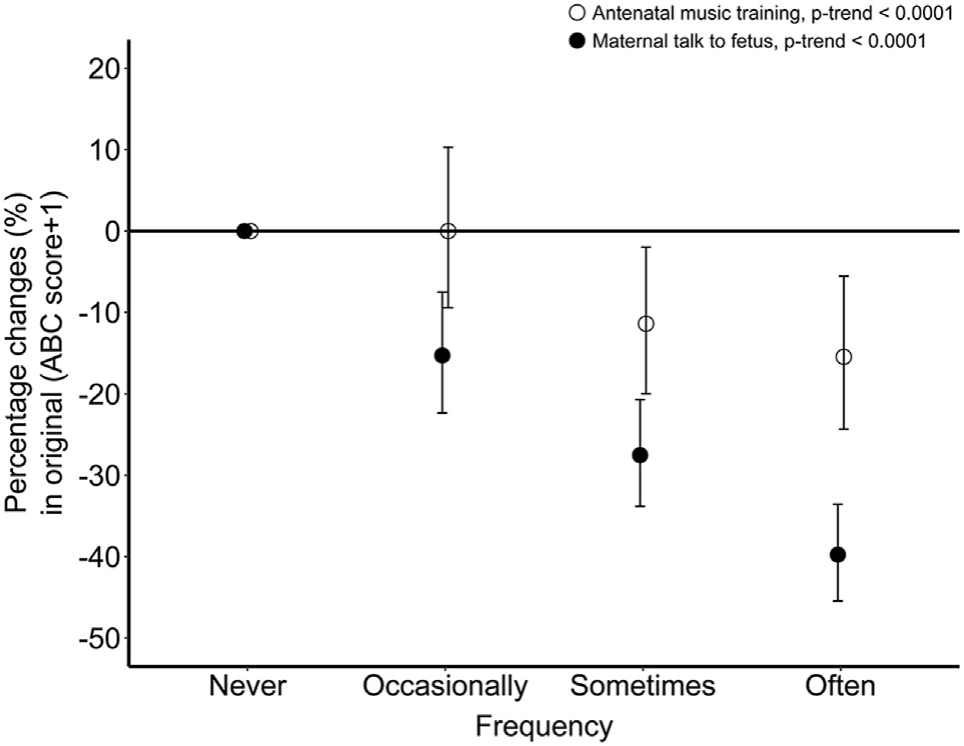
The percentage change of “ABC score + 1” in association with different frequency of antenatal music training (white circles) and maternal talk to fetus (black circles). Percentage changes were obtained by exponentiating the natural log scaled beta coefficient of Tobit regression and subtracting 1. Values were adjusted for family income, children’s sex and mother’s marital status, education level, age at birth of the study child, and gestational diseases. ABC, autism behavior checklist.

Furthermore, coefficients of Tobit regression were converted into the percentage changes of “ABC score + 1” in different frequency groups of antenatal music training and maternal talk to fetus, which are displayed in Figure 1. The percentage change of “ABC score + 1” increased with the increasing frequency of both antenatal music training and maternal talk to fetus, but it was larger in children who experienced maternal talk than in children who experienced antenatal music training.

In terms of the subscale scores, the same pattern of findings can be found for all of the subscales with the exception of antenatal training on Body and Object Use. For this subscale, there was no difference across the frequency of antenatal music training, although there still was a difference across the frequency of maternal talk for the fetus. The size of the effects appeared to be consistently stronger for maternal talk to fetus than that for antenatal music training, with such effect being the strongest for the Relating and Language subscales.

### Interaction between Antenatal Music Training and Maternal Talk to Fetus on Autistic-Like Behaviors around the Age of 3 Years

A two-way factorial ANCOVA on the total ABC scores indicated that the main effect of maternal talk to fetus on autistic-like behaviors was significant (Table 4, *F*-value = 5.83, *p* = 0.0006) and that there was a significant interaction between antenatal music training and maternal talk to fetus on autistic-like behaviors was also observed (Table 4, *F-*value = 11.87, *p* < 0.0001). This finding was replicated in all of the subscale scores (see Table 4 below).

**Table 4:** Main and interaction effects of antenatal music training and maternal talk to fetus.^a^

Source of variation	Degree of freedom	Sums of squares	Mean square	*F*-value	*p*-Value
**Sensory**					
Antenatal music training	3	1.532	0.511	1.30	2.272
Maternal talk to fetus	3	4.224	1.408	3.59	0.013
Antenatal music training × maternal talk to fetus	9	14.822	1.647	4.20	<0.0001
Error	34,721	13,623.442	0.392		
Total	34,748	13,855.914			

**Relating**					
Antenatal music training	3	0.417	0.139	0.22	0.882
Maternal talk to fetus	3	16.102	5.367	8.52	<0.0001
Antenatal music training × maternal talk to fetus	9	32.629	3.625	5.75	<0.0001
Error	34,721	21,877.826	0.630		
Total	34,748	22,281.069			

**Body and object use**					
Antenatal music training	3	4.671	1.557	2.03	0.108
Maternal talk to fetus	3	4.216	1.405	1.83	0.139
Antenatal music training × maternal talk To fetus	9	56.338	6.260	8.15	<0.0001
Error	34,721	26,666.373	0.768		
Total	34,748	27,014.687			

**Language**					
Antenatal music training	3	2.404	0.801	1.48	0.218
Maternal talk to fetus	3	13.139	4.380	8.08	<0.0001
Antenatal music training × maternal talk to fetus	9	21.419	2.380	4.39	<0.0001
Error	34,721	18,812.788	0.542		
Total	34,748	19,382.333			

**Social and Self-help skills**					
Antenatal music training	3	1.936	0.645	0.87	0.456
Maternal talk to fetus	3	10.300	3.433	4.63	0.003
Antenatal music training × maternal talk to fetus	9	59.701	6.633	8.94	<0.0001
Error	34,721	25,763.865	0.742		
Total	34,748	26,366.647			

**Total ABC score**					
Antenatal music training	3	3.223	1.074	0.67	0.573
Maternal talk to fetus	3	28.199	9.400	5.83	0.0006
Antenatal music training × maternal talk to fetus	9	172.158	19.129	11.87	<0.0001
Error	34,721	55,967.490	1.612		
Total	34,748	57,491.926			

*^a^Analysis of covariance adjusted for family income, children’s sex and mother’s marital status, education level, age at birth of the study child, and gestational diseases. ABC, autism behavior checklist*.

**Figure 2:**
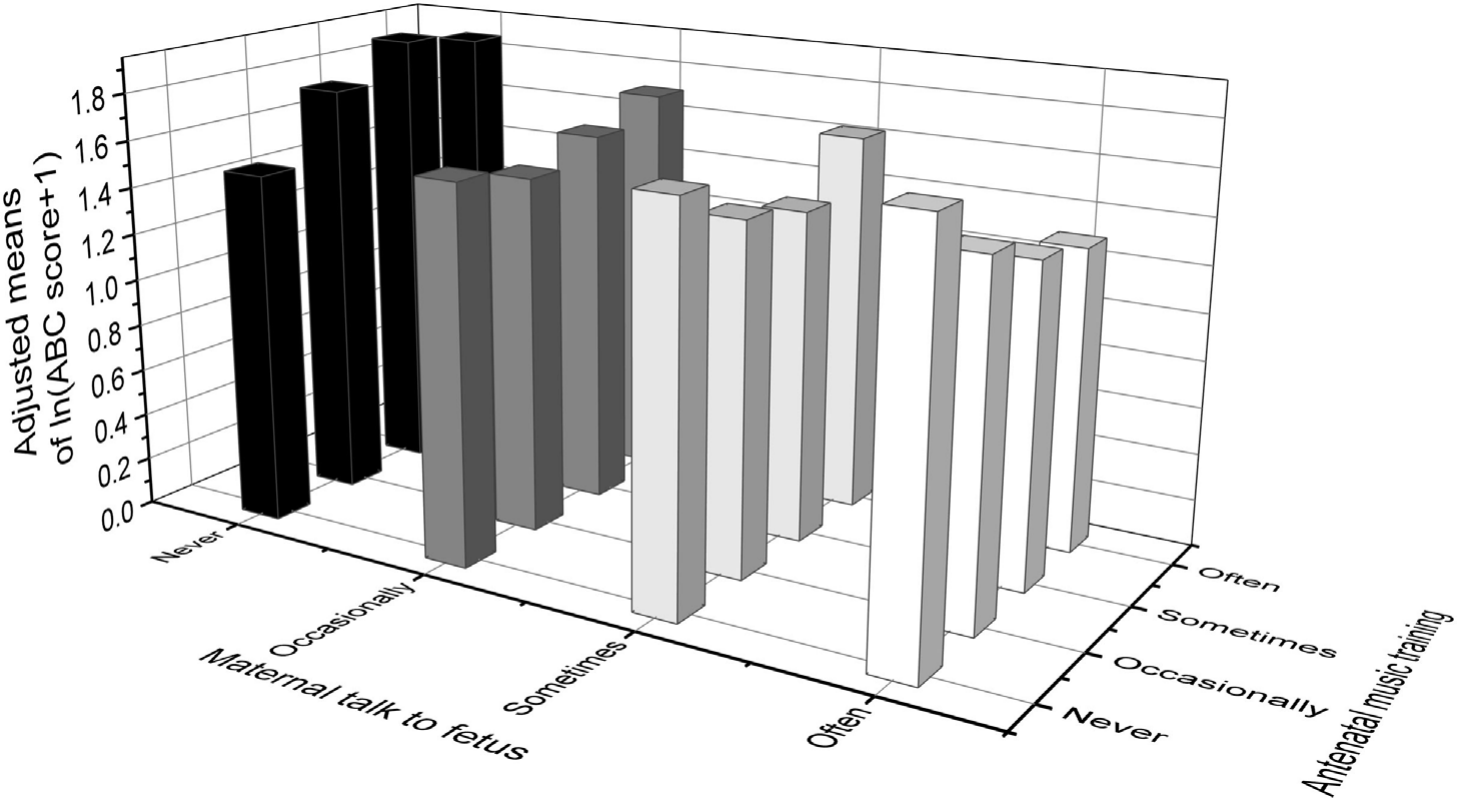
Adjusted means of ln(ABC score + 1) among different quantity of antenatal music training and maternal talk to fetus. Values were adjusted for family income, children’s sex and mother’s marital status, education level, age at birth of the study child and gestational diseases. ABC, autism behavior checklist.

Table 5 and Figure 2 show the adjusted means of natural log (ln) transformed “ABC score + 1” among different frequencies of antenatal music training and maternal talk to fetus, and two adjusted means that do not share a same lower-case letters were significantly different (at α < 0.05), according to *post hoc* multiple comparisons with Bonferroni correction.

**Table 5:** Adjusted means and SEs of ln(ABC score + 1) of children at around 3 years of age across different quantities of antenatal music training and maternal talk to fetus.^i^^,^^j^

Maternal talk to fetus	Antenatal music training			
	Never	Occasionally	Sometimes	Often
**Sensory**				
Never	0.412 (0.033)^a,b,c,d^	0.507 (0.034)^e^	0.535 (0.054)^a,b,e^	0.600 (0.110)^a,b,c,d,e^
Occasionally	0.485 (0.038)^a,e^	0.431 (0.029)^a^	0.438 (0.031)^a,b,e^	0.471 (0.041)^a,b,c,e^
Sometimes	0.521 (0.062)^a,b,c,d,e^	0.393 (0.030)^a,b,c,d^	0.391 (0.029)^b,c,d^	0.453 (0.033)^a,b,e^
Often	0.467 (0.094)^a,b,c,d,e^	0.391 (0.035)^a,b,c,d^	0.358 (0.032)^d^	0.364 (0.030)^c,d^

**Relating**				
Never	0.606 (0.042)^a,b^	0.724 (0.043)^c,d^	0.841 (0.069)^c^	0.841 (0.140)^a,b,c,d,e,f^
Occasionally	0.637 (0.048)^a,b,c,d^	0.598 (0.037)^a^	0.632 (0.039)^a,d^	0.637 (0.052)^a,b,c,d^
Sometimes	0.714 (0.079)^a,b,c,d,e,f^	0.591 (0.038)^a,b^	0.548 (0.037)^b,e^	0.629 (0.042)^a,b,c,d^
Often	0.490 (0.119)^a,b,c,d,e,f^	0.579 (0.045)^a,b,e,f^	0.490 (0.040)^e,f^	0.490 (0.039)^f^

**Body and object use**				
Never	0.668 (0.046)^a,b^	0.829 (0.047)^c^	0.924 (0.076)^c,d^	0.886 (0.154)^a,b,c,d^
Occasionally	0.767 (0.053)^a,b,c,d^	0.689 (0.040)^a^	0.766 (0.043)^b,c,d^	0.844 (0.057)^c,d^
Sometimes	0.718 (0.087)^a,b,c,d^	0.705 (0.042)^a,b,d^	0.686 (0.041)^a^	0.798 (0.046)^c,d^
Often	0.966 (0.131)^a,b,c,d^	0.684 (0.049)^a,b,d^	0.679 (0.044)^a,b^	0.638 (0.043)^a^

**Language**				
Never	0.583 (0.039)^a,b,c,d,e^	0.676 (0.039)^a,f^	0.805 (0.064)^f^	0.686 (0.129)^a,b,c,d,e,f,g,h^
Occasionally	0.594 (0.044)^a,b,c,d,e,f^	0.575 (0.034)^b,d^	0.611 (0.036)^a,b^	0.633 (0.048)^a,b,c,d,f^
Sometimes	0.649 (0.073)^a,b,c,d,e,f,g,h^	0.504 (0.035)^e,g,h^	0.513 (0.034)^c,e,g^	0.593 (0.039)^a,b,d^
Often	0.550 (0.110)^a,b,c,d,e,f,g,h^	0.496 (0.041)^c,d,e,g,h^	0.456 (0.037)^g,h^	0.452 (0.036)^h^

**Social and self-help skills**				
Never	0.817 (0.046)^a,b^	0.950 (0.046)^c^	1.062 (0.075)^c,d^	1.055 (0.152)^a,b,c,d,e,f^
Occasionally	0.861 (0.052)^a,b,c,d^	0.799 (0.040)^a^	0.837 (0.042)^a,d^	0.861 (0.056)^a,b,c,d^
Sometimes	0.914 (0.085)^a,b,c,d,e,f^	0.784 (0.041)^a,b,e^	0.740 (0.040)^b,e^	0.857 (0.046)^a,c,d^
Often	0.976 (0.129)^a,b,c,d,e,f^	0.795 (0.049)^a,b,e^	0.704 (0.044)^e,f^	0.653 (0.042)^f^

**Total ABC score**				
Never	1.493 (0.067)^a,b,c,d^	1.770 (0.068)^e^	1.919 (0.110)^e,f^	1.861 (0.223)^a,b,c,d,e,f,g^
Occasionally	1.591 (0.077)^a,b,e,f^	1.512 (0.059)^a,b^	1.602 (0.062)^a,f^	1.701 (0.083)^a,e,f^
Sometimes	1.663 (0.126)^a,b,c,d,e,f,g^	1.474 (0.061)^b,c^	1.406 (0.059)^c,d^	1.623 (0.067)^a,e,f^
Often	1.732 (0.190)^a,b,c,d,e,f,g^	1.479 (0.072)^a,b,c,d^	1.351 (0.064)^d,g^	1.291 (0.062)^g^

*^a–h^Two adjusted means that share one or more of the same lower-case letters are not significantly different at α = 0.05, after applying the multiplicity adjustment with Bonferroni correction. Abbreviations as in Table 3*.

*^i^Analyses of covariance adjusted for family income, children’s sex, and mother’s marital status, education level, age at birth of the study child and gestational diseases*.

*^j^Adjusted means are presented on the natural log scale, and they can also be interpreted as percentage differences between different groups by exponentiating the means and taking the ratio*.

For the mothers who never or only occasionally spoke with their fetus during pregnancy, there was trend for an increase in the ABC total scores along with an increase in the frequency of antenatal music played to the fetus. In contrast, for the mothers who often spoke with their fetus, there was a linear trend for a decrease in the ABC total scores along with an increase in the frequency of antenatal music played to the fetus. For the mothers who sometimes talked with the fetus, there was a slight U-shaped trend in the ABC scores with increases in frequency of antenatal music played. Alternatively, for mothers who never played music to their fetus, there was a linear trend of an increase in ABC total scores (but not statistically significant) along with an increase in the frequency of maternal talk; while in contrast, for mothers who played music occasionally, sometimes and often to their fetus, there was a linear decline in ABC total scores as the frequency of maternal talk to the fetus increased. This pattern of results was largely replicated in each of the subscale scores.

## Discussion

### Main Results

Our study used baseline information of the LCCS to investigate the associations of antenatal music training and maternal talk to fetuses during pregnancy upon autistic-like behaviors in children aged about 3 years. Our Tobit analyses showed that both antenatal music training and maternal talk to fetuses were protective factors against the development of autistic-like behaviors. Meanwhile, factorial ANCOVA found an interaction effect between antenatal music training and maternal talk to fetuses on autistic-like behaviors, with autistic-like behaviors being lowest in children who often experienced both antenatal music training and maternal talk when a fetus. In particular, antenatal music training appeared to be associated with a significant increase in autistic-like behaviors among children who never or only occasionally experienced maternal talk as a fetus, but was associated with a reduction in autistic-like behaviors among those often experienced maternal talk. Moreover, an increasing frequency of maternal talk to the fetus was associated with a significantly decreased risk of the autistic-like behaviors among children who were occasionally or sometimes or often exposed to antenatal music training, while it was associated with an increased risk of autistic-like behaviors among children never experienced antenatal music training. While these trends were present, statistical differences between the means in the cells of the Table 5 displaying total ABC scores were not always present. This was at least partly due to the relatively small sample sizes and the larger SDs found in the Never-Often (i.e., Never Maternal Talk and Often Antenatal Music, or the Often Maternal Talk and Never Antenatal Music) and surrounding cells. As such any detailed level of analysis and interpretation of the data in Table 5 needs to be done with caution. However, these data do help clarify the nature of the interaction effects displayed in Table 4.

A further interesting finding from this study was that in addition to finding main effects and an interaction effect for total ABC scores, the same pattern of results was observed for the five subscales of the ABC. Our finding that exposing a fetus to music and maternal speech during pregnancy is associated with a broad reduction in autistic-like behaviors is congruent with previous findings of music training for children with ASD. A Cochrane review has indicated that music therapy significantly improves a wide range of ASD symptoms in children with ASD including social interaction, verbal communication, initiating behavior, social–emotional reciprocity, as well as social adaptation skills (44). Similarly, there is emerging evidence that the style of maternal talk to her child influences not only verbal development in ASD children (45), but also ASD children’s cognitive development in other areas such as the development of the child’s theory of mind (46). This is a helpful finding indicating that the simple and cost-effective intervention of talking with a fetus and playing music with a fetus may have a broad positive impact upon the symptoms of ASD displayed by a child after he/she is born. This finding also supports a broad neuroanatomical mechanism linking these two maternal behaviors with the reduced rate of ASD symptoms in young children.

### Possible Mechanisms

Our study found an inverse association between antenatal music training and autistic-like behaviors in young children, which is in accordance with previous studies that children’s functional development may be associated with prenatal experience of music (47). Moreover, a randomized controlled trial study found that antenatal music exposure could influence the fetal behavior state and this effect might carry over into the neonatal period (48). Recently, researchers have attempted to reveal the underlying mechanisms of how antenatal music training favorably affects neurobehavioral development. For example, a study by Kim et al. (49) found that prenatal music stimulation could enhance a rat’s brain development and spatial learning ability by increasing the neurogenesis of hippocampus and some layers in the motor and matosensory cortex. Another study by Chikahisa et al. (50) revealed that music stimulation enhanced mice’s brain functions by improving their synaptic plasticity through regulating BDNF/TrkB signaling and its intracellular signaling pathway targets. Furthermore, two studies, respectively, showed that prenatal exposure to music could greatly affect the brain neuroplasticity and that these effects were long lasting in mice models (51) as well as in humans (52).

In addition to music, there is also emerging evidence that maternal talk to a fetus can influence neurological development. For example, Fifer and Moon (53) found that mother’s voice plays an important role in the organization of brain function, and Krueger and Garvan (54) observed that maternal talk during pregnancy might improve children’s early speech. Taken together, these studies indicate that both music and maternal talk to a fetus may improve a fetus’ or child’s neurobehavioral development.

### Meaning of the Statistical Interaction between Music Training and Talking to Fetus

The additional finding of an interaction effect is an important development upon previous research findings of main effects for maternal talking and music playing for human fetus development. Our study has found that it is common for pregnant women in China (89.87%) to both talk to and play music to their fetus while pregnant. However, the interaction effect of these two behaviors has not been previously investigated, because prior studies have only assessed either one of these maternal behaviors (24, 55, 56). Our study implies that the sole action of antenatal music training or maternal talk to fetus may be relatively harmful to children’s neurobehavioral development, while the combination of antenatal training with both music and maternal talk to fetus may be beneficial. As such our findings suggest that it may be important for future studies to include measures of both maternal behaviors toward their unborn fetuses to explore the possibility of such interaction effects.

The finding of an interaction effect begs the question of why there may be an interaction effect above and beyond the main effects of maternal talking to the fetus and the playing of music to the fetus? One possible explanation is that this represents a mere increased dosage of a common underlying mechanism upon cognitive development. For example, a study by Webb et al. (57) explored the impact of audio recordings of maternal sounds (mother’s voice and heart beat) on the development of the auditory cortex in extremely premature infants. The researchers found that the newborns exposed to the maternal sounds had a larger auditory cortex compared with controls after the first month. What makes this study particularly interesting was that the recordings of the concurrent mother’s voice and heart beat were first exposed to a low pass filter prior to presentation to the premature newborn to mimic the muffled sounds that an unborn fetus was likely to hear. Given that the filtering interrupted the intelligibility of individual syllables, this made prosody the primary acoustic element. That is, perhaps it was the prosodic features such as melody, intensity, and rhythm of the mother’s heart beat and voice that resulted in different structural patterns of brain maturation. Such prosodic features of course parallel the same characteristics of melody, intensity, and rhythm in music. Supporting such a hypothesis are findings that exposure to music *in utero* is also associated with neuroplasticity in the auditory cortex (58, 59).

Another possible explanation for the interaction effect is that the degree of not speaking to the fetus or not playing music to the fetus may be proxy measures of the presence of a psychiatric disorder or even ASD symptoms in the mother. That is, perhaps mothers who are very empathic or less affected by a psychiatric disorder, are more likely to both speak to their fetus and play music to their unborn fetus. In contrast, perhaps mothers who do not speak to their fetus or play music to their fetus have higher rates of ASD symptoms or psychiatric symptoms. Such a possibility presents at least two causal hypotheses that need to be explored further in future studies. First is genetic heritability. That is, perhaps mothers who do not play music and speak with the fetus may have symptoms of ASD or a psychiatric illness that is a genetic risk factor for the presence of ASD symptoms in their child, while mothers who both play music and speak with their fetus may have genetic dispositions that are protective against ASD symptoms in their child. Such a hypothesis is supported by estimates of 38% heritability in ASD (60) and evidence that parental psychiatric history is a risk factor for ASD (61). A second but associated hypothesis is that a mother’s symptoms of ASD or presence of psychiatric disorder may influence her decision over the frequency and type of music and speech given to the fetus, and that this frequency and type of music and speech may have a causal influence upon the risk of ASD symptoms in the child. For example, there is emerging evidence that empathic individuals and systemizing individuals have quite different preferences in music (62). This is important given that measures of empathizing and systemizing predict scores on the Autistic Spectrum Quotient (63). As such, individuals high in ASD symptoms are likely to choose music with different qualities, or speak to their fetus with different prosodic qualities, than individuals low in ASD symptoms. This is an important hypothesis to explore further, given that there are some indicators that the type of music and instrument played may moderate the link between music training and neuroplasticity (64). It is, therefore, possible that mothers who are highly empathic may choose music or speech that they believe will be soothing or uplifting for the child. In contrast, mothers who do not speak with their fetus (which might be a marker for low empathy) and who frequently play music to their fetus, may choose music that is stimulating to them but may be a risk factor for ASD symptoms in the child.

### Limitations and Future Directions

Although our findings are encouraging, several limitations should be considered. First, it was a cross-sectional study that used the baseline information of the LCCS; therefore, we could not determine the casual relationship of the antenatal training of music stimulation and maternal talk to the fetus with autistic-like behaviors at 3 years of age. Second, the data were retrospectively collected through a structured questionnaire completed by the children’s primary caregivers, which might lead to an informant bias. Third, we did not collect detailed information on antenatal training, such as the method of antenatal music training (e.g., putting on abdomen or not), the frequency in different trimesters of pregnancy, and the music type. The omission of such information prevented us from obtaining a more sophisticated understanding of the moderating variables linking effects of antenatal music training and maternal talk to a fetus with autistic-like behaviors in young children. Fourth, music stimulation and maternal talk to fetus might continue even after child birth, which could also affect autistic-like behaviors at around 3 years of age, unfortunately, this study did not control for these effects when we evaluated the relationship of the antenatal training of music stimulation and maternal talk to fetus with autistic-like behaviors.

As such there is a need to replicate the findings of this study through longitudinal and interventional studies that include more detailed measures of the qualities and characteristics of the maternal talk and music played to fetuses in Chinese pregnant women. If our findings are replicated in future studies, then they will provide important evidence to support a relatively simple, low-cost, and sustainable public health intervention to reduce the rates of autistic-like behaviors in children. Such an intervention could have a profound positive impact upon reducing the emotional, social, and economic burden on individuals, families, and societies affected by autistic-like behaviors.

## Conclusion

Taken together, our results suggest that antenatal training through playing music and maternal talk to the unborn fetus are associated with a reduced risk of autistic-like behaviors in children at around 3 years of age. Moreover, the findings from this study indicate an interaction effect between the frequencies of playing music and talking to a fetus, and show that fetuses who often experience both antenatal music training and maternal talk have the lowest risk of autistic-like behaviors as a 3-year-old child. These findings support emerging evidence for the need of public health interventions to encourage pregnant mothers to frequently talk with their fetus as well as play music to the fetus, in order to reduce the risk of autistic-like behaviors in young children.

## Ethics Statement

This study was approved by the Ethic Committee of School of Public Health of Sun Yat-sen University. Written informed consent was obtained from all parents or guardians of children who took part in the study, in accordance with the Declaration of Helsinki. The protocol was approved by the Ethic Committee of School of Public Health of Sun Yat-sen University.

## Author Contributions

W-QC, C-AW, LL, Z-LR, HJ, JJ, YJ, and G-MW initiated and designed the study. W-QC led the research training. C-AW led the field investigation and LL, Z-LR, X-NY, D-LS, and D-XX took part in the investigation team. Z-LR, W-QC, and ES analyzed and interpreted the data. Z-LR and W-QC wrote the manuscript, ES and L-JF revised it with suggestions from all authors.

## Conflict of Interest Statement

The authors declare that the research has been conducted in the absence of any commercial or financial relationships that could be construed as a potential conflict of interest.

## Abbreviations

ASD, autism spectrum disorder; LCCS, Longhua child cohort study; ABC, autism behavior checklist.
